# Supplementary material for: Spatial organization of adenylyl cyclase and its impact on dopamine signaling in neurons
Source: Nat Commun. 2024 Sep 27;15:8297. doi: 10.1038/s41467-024-52575-0 (PMC11436756; doi:10.1038/s41467-024-52575-0)
Supplement: Supplementary file 1 — Supplementary Information [file 41467_2024_52575_MOESM1_ESM.pdf]

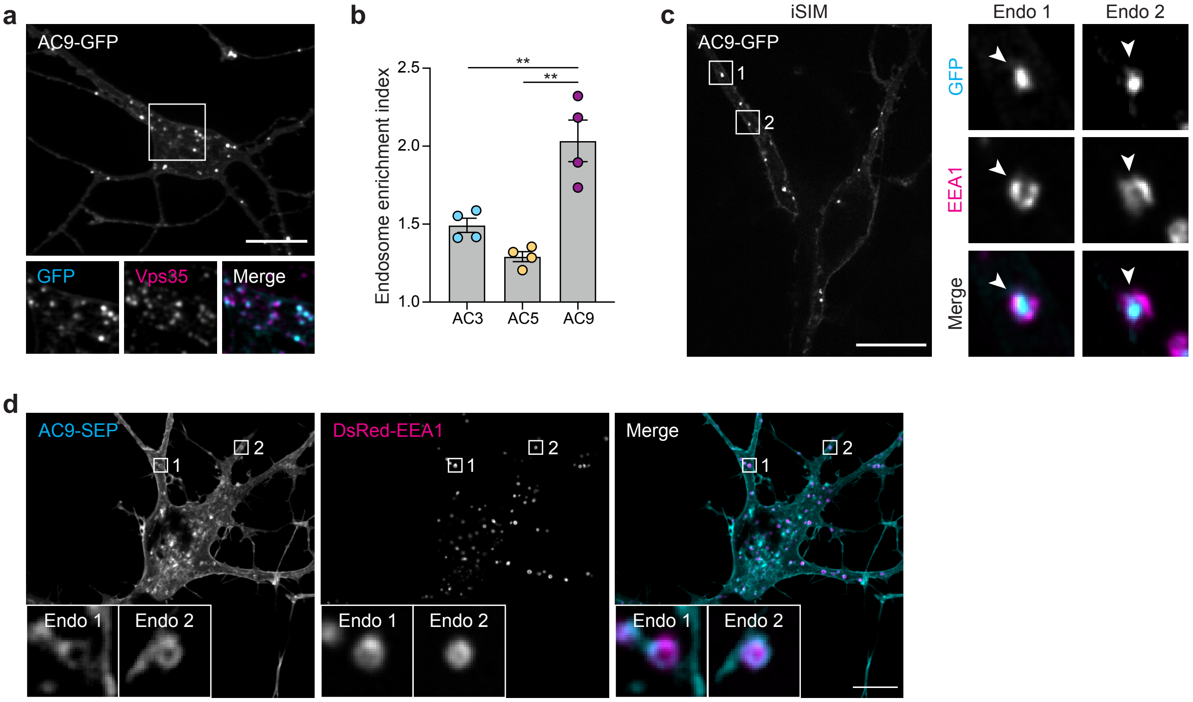

Supplementary Fig 1. AC9 localizes to endosomes. a, Maximum intensity Z-projection of confocal microscopy images of MSNs expressing AC9-GFP and stained for endosomal marker Vps35. **b,** Endosome enrichment index calculated as a ratio of AC-GFP fluorescence intensity at Vps35 positive endosomes divided by total cell fluorescence. Data are shown as mean ± s.e.m from *n* = 4 independent experiments (37-47 cells total/condition). ***P* = 0.0085 for AC3 vs AC9, ***P* = 0.0017 for AC5 vs AC9 by unpaired two-tailed Student's *t*-test. **c,** Representative iSIM images of MSN expressing AC9-GFP and stained for EEA1. Arrowheads indicate the endosomal membrane. **d**, Representative live cell spinning disk confocal images of MSN coexpressing AC9-SEP and Ds-Red-EEA1. Data represent biological replicates and are shown as individual data points or mean ± s.e.m. Scale bars are 10 μm. Source data are provided as a Source Data file.

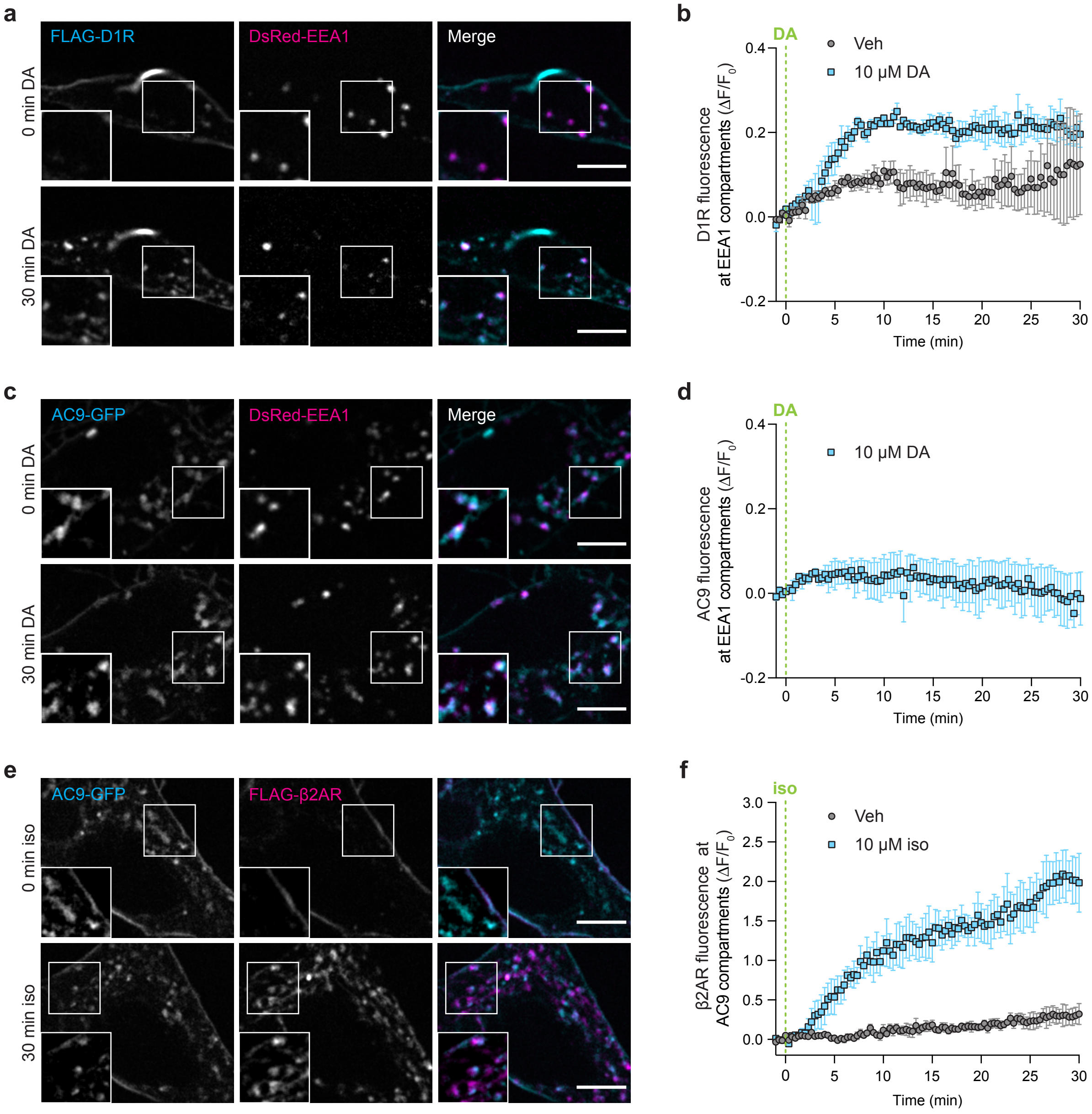

Supplementary Fig 2. Dopamine 1 receptor and AC9 localize to EEA1 positive endosomes. a, Representative live cell spinning disk confocal images of MSNs transfected with FLAG-D1R and DsRed-EEA1 and treated with 10 μM dopamine (DA) at 0 min. Surface FLAG-D1R was labeled with Alexa Fluor 555-coupled anti-FLAG antibody for 15 min before imaging. **b,** Quantification of surface labeled FLAG-D1R accumulation at segmented EEA1-positive endosomes after vehicle (Veh) or 10 μM DA addition. Data are shown as mean ± s.e.m from *n* = 3 independent experiments (26-27 cells total/condition). **c,** Representative live cell spinning disk confocal images of MSNs transfected with AC9-GFP and DsRed-EEA1 and treated with 10 μM DA at 0 min. **d,** Quantification of AC9-GFP accumulation at segmented EEA1-positive endosomes after 10 μM DA addition. Data are shown as mean ± s.e.m from *n* = 3 independent experiments (23 cells total/condition). **e**, Representative live cell spinning disk confocal images of MSNs transfected with FLAG-β2AR and AC9-GFP and treated with 10 μM isoproterenol (iso) at 0 min. Surface FLAG-β2AR was labeled with Alexa Fluor 555-coupled anti-FLAG antibody for 15 min before imaging. **f**, Quantification of surface labeled FLAG-β2AR accumulation at segmented AC9-positive endosomes after vehicle (Veh) or 10 μM iso addition. Data are shown as mean ± s.e.m from *n* = 3 independent experiments (22-23 cells total/condition). Scale bars are 5 μm. Source data are provided as a Source Data file.

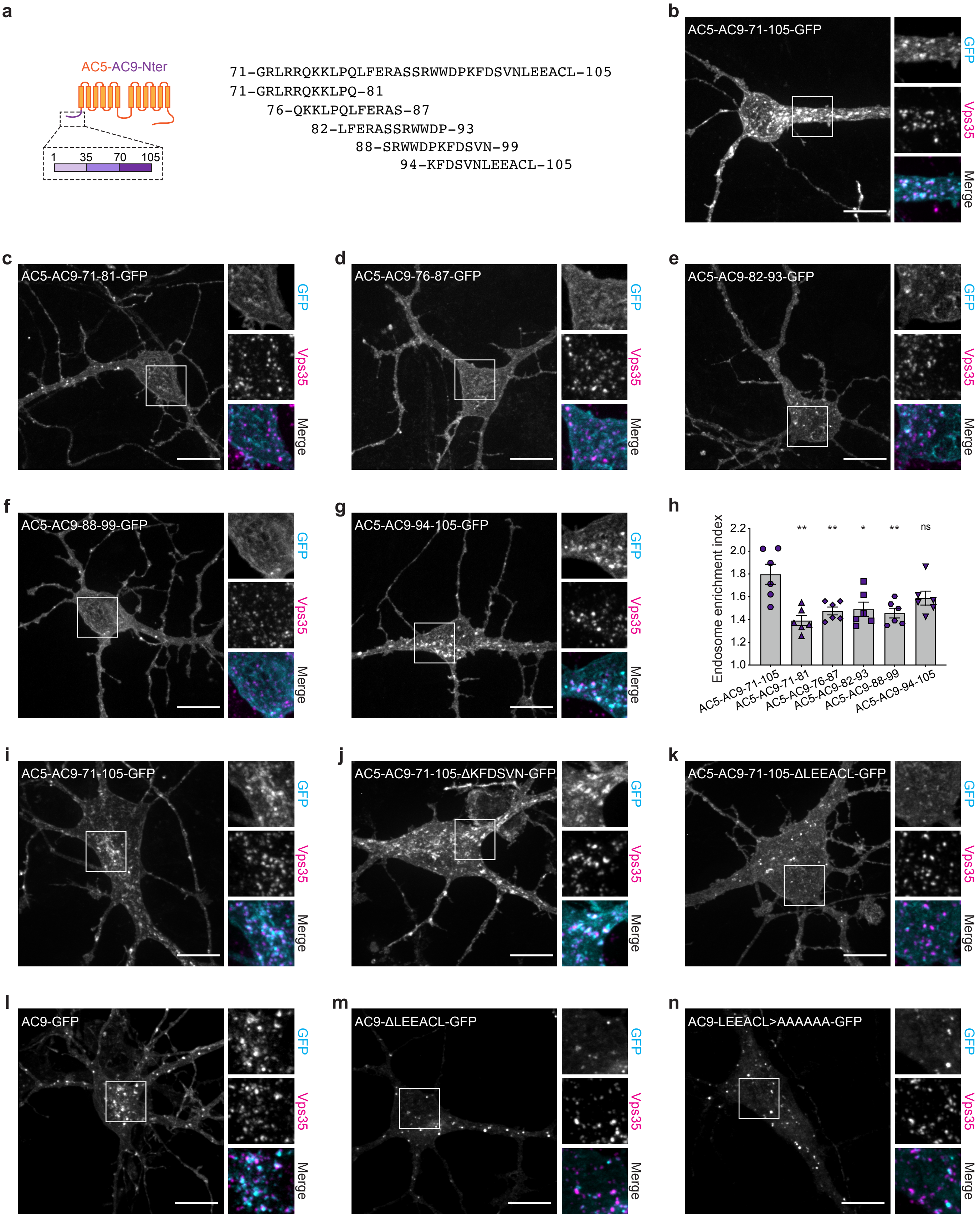
a
AC5-AC9-Nter
1 35 70 105
71-GRLRRQKKLPQLFERASSRWWDPKFDSVNLEEACL-105
71-GRLRRQKKLPQ-81
76-QKKLPQLFERAS-87
82-LFERASSRWWDP-93
88-SRWWDPKFDSVN-99
94-KFDSVNLEEACL-105
b
AC5-AC9-71-105-GFP
GFP
Vps35
Merge
c
AC5-AC9-71-81-GFP
d
AC5-AC9-76-87-GFP
e
AC5-AC9-82-93-GFP
f
AC5-AC9-88-99-GFP
g
AC5-AC9-94-105-GFP
h
Endosome enrichment index
2.2
2.0
1.8
1.6
1.4
1.2
1.0
** ** * ** ns
AC5-AC9-71-105
AC5-AC9-71-81
AC5-AC9-76-87
AC5-AC9-82-93
AC5-AC9-88-99
AC5-AC9-94-105
i
AC5-AC9-71-105-GFP
j
AC5-AC9-71-105-ΔKFDSVN-GFP
k
AC5-AC9-71-105-ΔLEEACL-GFP
l
AC9-GFP
m
AC9-ΔLEEACL-GFP
n
AC9-LEEACL>AAAAAA-GFP

Supplementary Fig 3. A second motif in the N-terminus of AC9 is required for endosome localization. a, Schematic representation of AC5-AC9-Nter and the sequences of the distal portion of AC9 N-terminus (71-105 amino acids) used for the chimeric mutants of AC5. **b-g**, Maximum intensity Z-projection of confocal images microscopy images of MSNs expressing AC5-AC9-71-105-GFP (**b**), AC5-AC9-71-81-GFP (**c**), AC5-AC9-76-87-GFP (**d**), AC5-AC9-82-93-GFP (**e**), AC5-AC9-88-99-GFP (**f**) or AC5-AC9-94-105-GFP (**g**) and stained for the endosomal marker Vps35. **h**, Endosome enrichment index of cells expressing the different chimeric mutants of AC5. Data are shown as mean ± s.e.m from n = 6 independent experiments (≥30 cells total/condition). **P = 0.002 for AC5-AC9-71-81 vs AC5-AC9-71-105, **P = 0.0067 for AC5-AC9-76-87 vs AC5-AC9-71-105, **P = 0.0059 for AC5-AC9-88-99 vs AC5-AC9-71-105, *P = 0.0173 by unpaired two-tailed Student's t-test. **i-n**, Maximum intensity Z-projection of confocal images microscopy images of MSNs expressing AC5-AC9-71-105-GFP (**i**), AC5-AC9-71-105-ΔKFDSVN-GFP (**j**), AC5-AC9-71-105-ΔLEEACL-GFP (**k**), AC9-GFP (**l**), AC9-ΔLEEACL-GFP (**m**) or AC9-LEEACL>AAAAAA-GFP (**n**) and stained for the endosomal marker Vps35. Scale bars are 10 μm. Source data are provided as a Source Data file.

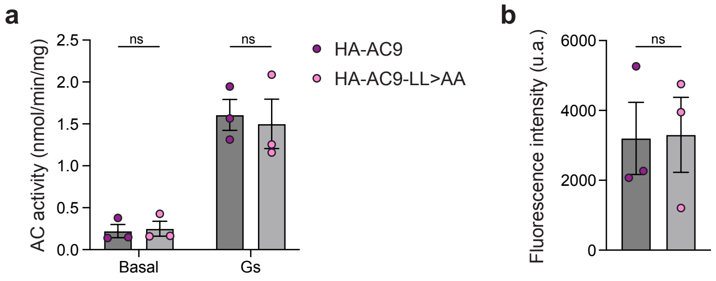

Supplementary Fig 4. AC9 and AC9-LL>AA show similar AC activity and expression levels. a, Membrane preparations from HEK293 cells expressing HA-AC9 and HA-AC9-LL>AA were subjected to AC enzyme assays for basal and Gα s-stimulated AC activity. Data are shown as mean ± s.e.m from *n* = 3 independent experiments. **b**, Quantification of the expression level by measuring HA fluorescence intensity of MSNs expressing HA-AC9 or HA-AC9-LL>AA. Data are shown as mean ± s.e.m from *n* = 3 independent experiments (30-31 cells total/condition). n.s. not significant by unpaired two-tailed Student's *t*-test. Source data are provided as a Source Data file.

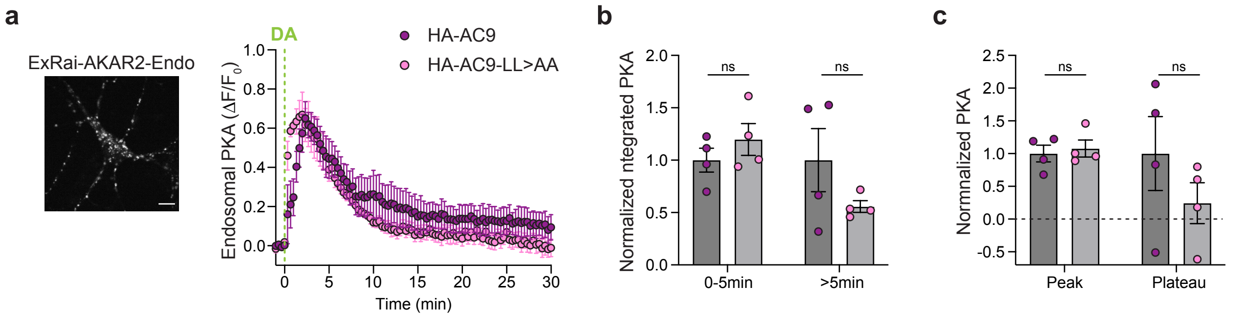

Supplementary Fig 5. Impairing AC9 endosomal localization impacts PKA activity at the endosomes. a, On the left, spinning-disk confocal representative image showing ExRai-AKAR2-Endo localization at the endosomes (Scale bar = 10 μ m). On the right, kinetics of PKA activity at endosomes over time in MSNs coexpressing ExRai-AKAR2-Endo and HA-AC9 (purple) or HA-AC9-LL>AA (pink), from *n* = 4 independent experiments (41-42 cells total/condition) and treated with 10 μM DA. The ΔF/F0 was measured every 20 sec and normalized to the Fsk and IBMX response. **b**, Integrated PKA signals of the phases 0-5 min and >5 min (5-30 min) after DA addition were calculated as the area under the curve and normalized to the average HA-AC9 value, from *n* = 4 independent experiments. **c**, Peak and plateau values were calculated as the maximum ΔF/F0 (peak) and the average of 20-30 min values (plateau) and were normalized to the HA-AC9 value, from *n* = 4 independent experiments. For all panels, data represent biological replicates and are shown as mean ± s.e.m. n.s. not significant by unpaired two-tailed Student's *t*-test. Source data are provided as a Source Data file.

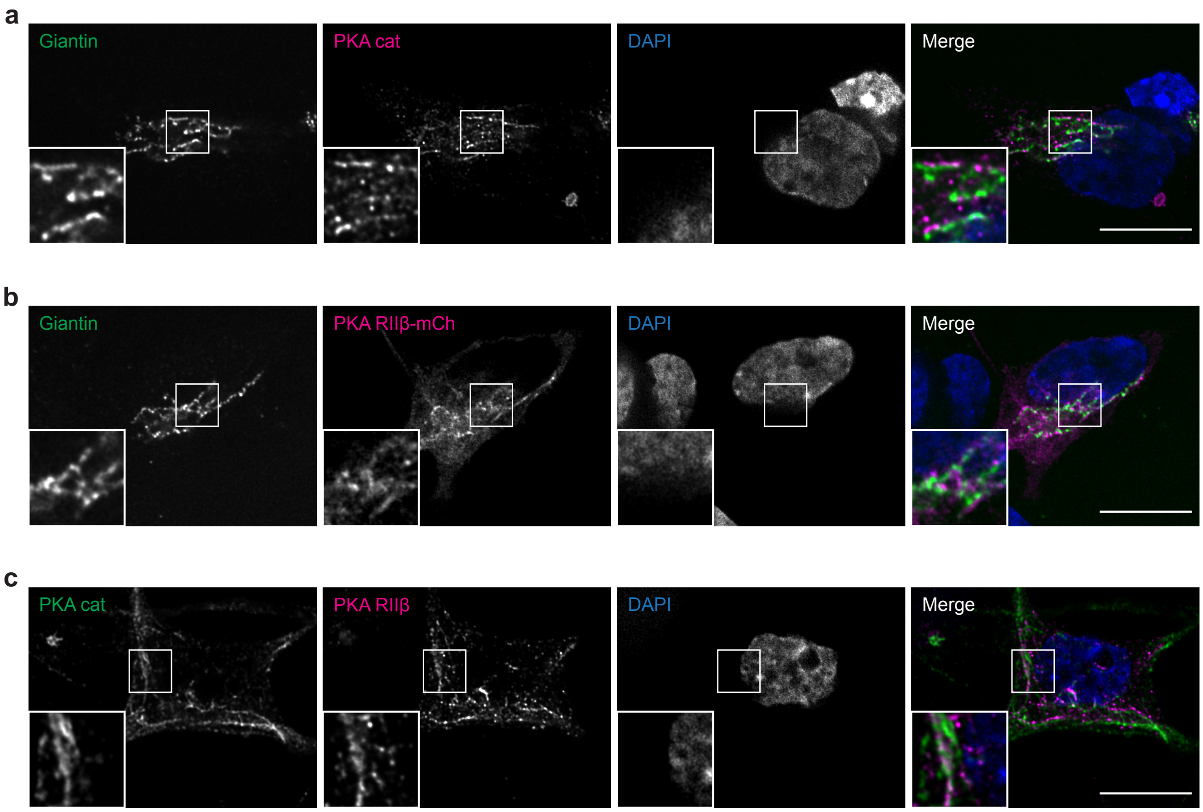

Supplementary Fig 6. PKA subunits localization at the Golgi. a, Representative iSIM images of MSN stained for the Golgi marker giantin and endogenous PKA cat. **b,** Representative iSIM images of MSNs expressing PKA RIIβ-mCh and stained for giantin. **c,** Representative iSIM images of MSN stained for endogenous PKA cat and PKA RIIβ. Nuclei were stained with DAPI. Scale bars are 10 μm.

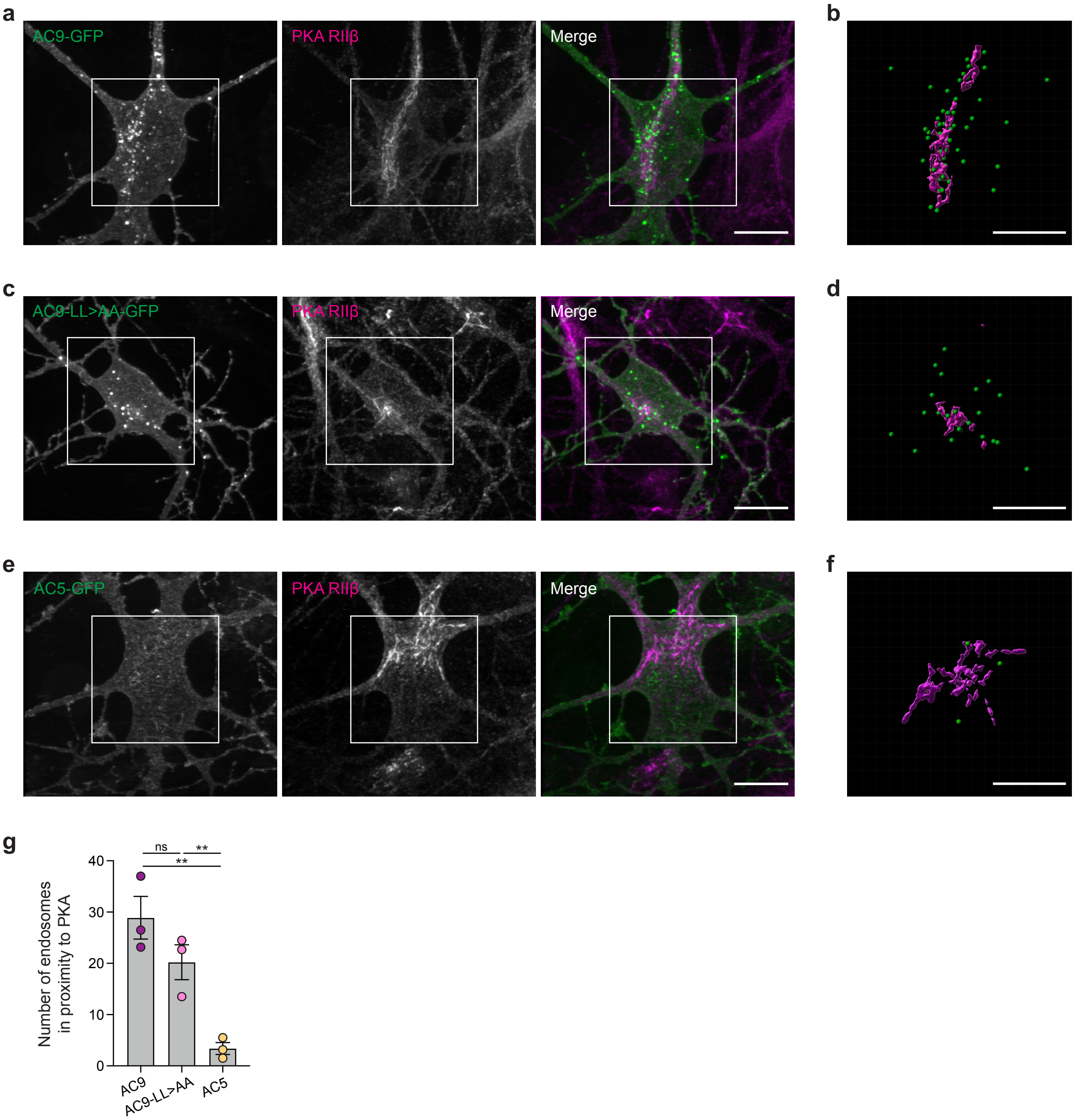

Supplementary Fig 7. AC9-positive endosomes are in close proximity with PKA. **a**,**c**,**e**, Maximum intensity Z-projection of confocal microscopy images of MSNs expressing AC9-GFP (**a**), AC9-LL>AA-GFP (**c**) or AC5-GFP (**e**) and stained for endogenous PKA RIIβ. **b**,**d**,**f**, 3D rendering of cells in **a** (**b**), **c** (**d**) and **e** (**f**). **g**, Quantification of the number of AC-positive endosomes colocalizing with PKA RIIβ. Data are shown as mean ± s.e.m from n = 3 independent experiments (18 cells total/condition). **P = 0.0041 for AC9 vs AC5, **P = 0.0094 for AC9-LL>AA vs AC5 by unpaired two-tailed Student's t-test. Scale bars are 10 μm. Source data are provided as a Source Data file.

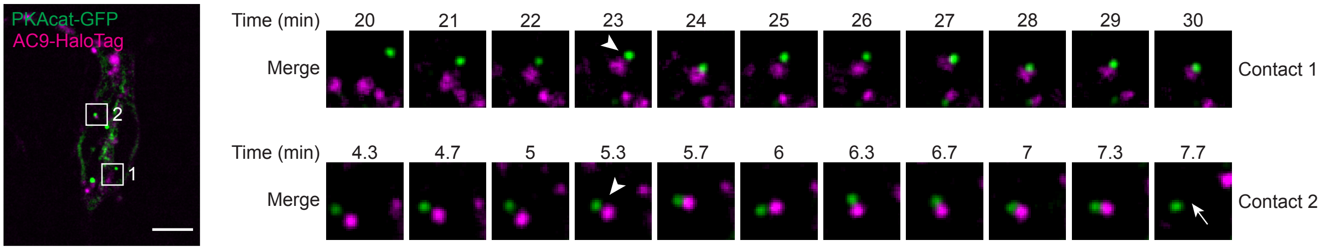

Supplementary Fig 8. Dynamic contacts between PKA cat puncta and AC9 containing endosomes. Spinning-disk confocal images from a time series of neurons expressing PKAcat-GFP and AC9-HaloTag and treated with 10 μM DA at t = 0 min, from *n* = 3 independent experiments. PKA cat puncta and AC9-containing endosomes form close contacts (arrowheads) for several minutes then separate (arrow). Scale bar is 5 μm.

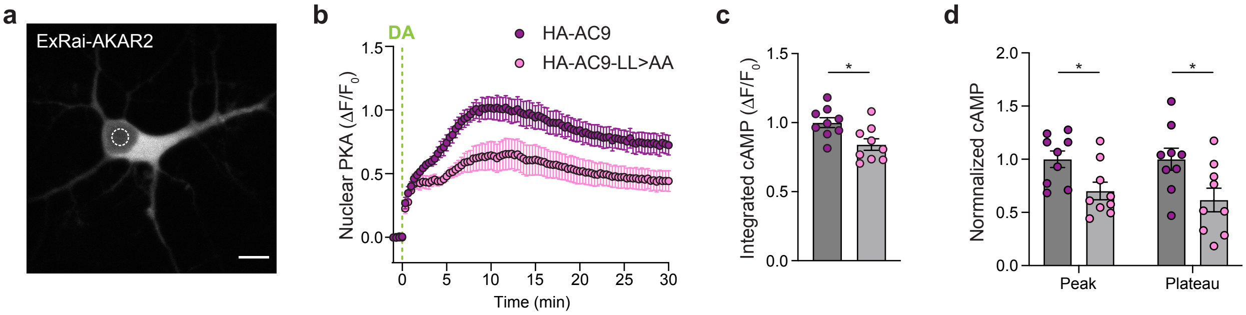

Supplementary Fig 9. Mutating AC9 dileucine motif impacts PKA activity in the nucleus. a, Spinning-disk confocal representative image of MSN expressing ExRai-AKAR2. A ROI is drawn inside the nucleus (white dotted circle) to measure PKA activity within the nucleus over time. Scale bar = 10 µm. **b,** Kinetics of nuclear PKA activity over time in MSNs coexpressing ExRai-AKAR2 and HA-AC9 (purple) or HA-AC9-LL>AA (pink) and treated with 10 µM dopamine (DA), from *n* = 9 independent experiments (120-126 cells total/condition). **c,** Integrated nuclear PKA signal was calculated as the area under the curve and normalized to the average HA-AC9 value, from *n* = 9 independent experiments. *P = 0.011 by unpaired two-tailed Student's *t*-test. **d,** Peak and plateau values were calculated as the maximum ΔF/F0 (peak) and the average of 20-30 min values (plateau) and normalized to the HA-AC9 value, from *n* = 9 independent experiments. *P = 0.0179 for Peak, *P = 0.0233 for Plateau by unpaired two-tailed Student's *t*-test. Data represent biological replicates and are shown as mean ± s.e.m. Source data are provided as a Source Data file.

Supplementary Table 1. List of chemicals used in this study

Chemical	Manufacturer	Catalog #	Solvant	Concentration
Forskolin	Millipore Sigma	F6886	DMSO	10 μM
IBMX	Millipore Sigma	I5879	DMSO	500 μM
Dopamine hydrochloride	Millipore Sigma	H8502	H_2O	10 μM
Cytosine arabinosine	Millipore Sigma	C6645	H_2O	2 μM

Supplementary Table 2. List of antibodies used in this study

Antibody	Manufacturer	Catalog #	Dilution
Rabbit anti-Arl13b	Proteintech	17711-1-AP	1:500
Rat anti-HA	BioLegend	901513	1:500
Mouse anti-EEA1	BD Biosciences	610457	1:500
Goat anti-Vps35	Novus Biologicals	NB100-1397	1:500
Mouse anti-FLAG M1	Millipore Sigma	F3040	NA
Rabbit anti PKAα cat	Santa Cruz	sc-903	1:1000
Mouse anti-PKA RIIβ	BD Biosciences	610625	1:500
Mouse anti-Giantin	Abcam	ab37266	1:500
Donkey anti rat IgG AlexaFluor 488	Invitrogen	A-21208	1:500
Goat anti rat IgG AlexaFluor 488	Invitrogen	A-11006	1:500
Goat anti rat IgG AlexaFluor 647	Invitrogen	A-21247	1:500
Goat anti rabbit IgG AlexaFluor 647	Invitrogen	A-21245	1:500
Donkey anti mouse IgG AlexaFluor 555	Invitrogen	A-31570	1:500
Donkey anti mouse IgG AlexaFluor 647	Invitrogen	A-31571	1:500
Donkey anti goat IgG AlexaFluor 647	Invitrogen	A-21447	1:500
Donkey anti rabbit IgG AlexaFluor 555	Invitrogen	A-31572	1:500

Supplementary Table 3. List of plasmids used in this study

Plasmid	Source
HA-AC3	This study
HA-AC5	This study
HA-AC9	This study
AC3-GFP	This study
AC5-GFP	This study
AC9-GFP	This study
AC9-SEP	This study
FLAG-D1R	A. Ehrlich
DsRed-EEA1	Irannejad et al 2013
AC5-AC9-Nter-GFP	This study
AC5-Δter-GFP	This study
AC9-AC5-Nter-GFP	This study
AC5-AC9-1-35-GFP	This study
AC5-AC9-36-70-GFP	This study
AC5-AC9-71-195-GFP	This study
AC5-AC9-1-35-LL>AA-GFP	This study
AC9-LL>AA-GFP	This study
AC5-AC9-71-81-GFP	This study
AC5-AC9-76-87-GFP	This study
AC5-AC9-82-93-GFP	This study
AC5-AC9-88-99-GFP	This study
AC5-AC9-94-105-GFP	This study
AC5-AC9-71-105-ΔLEEACL-GFP	This study
AC5-AC9-71-105-ΔKFDSVN-GFP	This study
AC9-ΔLEEACL-GFP	This study
AC9-LEEACL>AAAAAA-GFP	This study
ExRai-AKAR2 (pCAG)	This study
ExRai-AKAR2-NLS	This study
ExRai-AKAR2-NES	This study
ExRai-AKAR2-Endo	This study
AC9-HaloTag	This study
PKAcat-GFP	A. Marley
PKA RIIb-mCh	G. Peng
