# Supplementary material for: Spatial organization of adenylyl cyclase and its impact on dopamine signaling in neurons
Source: Nat Commun. 2024 Sep 27;15:8297. doi: 10.1038/s41467-024-52575-0 (PMC11436756; doi:10.1038/s41467-024-52575-0)
Supplement: Supplementary file 3 — Description of Additional Supplementary Files [file 41467_2024_52575_MOESM3_ESM.pdf]

Description of Additional Supplementary Files

File name: Supplementary Movie 1
Description: Movie of the 3D rendering of MSN expressing AC9-GFP (green) stained for endogenous PKA cat (magenta) and stained with DAPI (blue) to label the nucleus. The video shows extensive regions of close apposition between AC9-containing endosomes and Golgi-associated PKA cat stores close to the nucleus.

File name: Supplementary Movie 2
Description: Movie of the 3D rendering of MSN expressing AC9-GFP (green) and stained for endogenous PKA RIIβ (magenta). Nucleus was stained with DAPI (blue). The video shows proximity between AC9-positive endosomes and Golgi-associated PKA RIIβ compartments next to the nucleus.

File name: Supplementary Movie 3
Description: Movie of a live confocal image series of MSN expressing PKAcat-GFP (green) and AC9-HaloTag (magenta) and treated with 10 μM dopamine (DA). The video shows PKA cat puncta and AC9-containing endosomes moving together for several minutes, indicating close contact. Scale bar, 1 μm.

File name: Supplementary Movie 4
Description: Movie of a live confocal image series of MSN expressing ExRai-AKAR2 and treated with 10 μM dopamine (DA) after 1 min. Dopamine addition led to increased PKA activity in the cytoplasm, followed by the nucleus. Scale bar, 50 μm.
