# Supplementary material for: Spatial organization of adenylyl cyclase and its impact on dopamine signaling in neurons
Source: Nat Commun. 2024 Sep 27;15:8297. doi: 10.1038/s41467-024-52575-0 (PMC11436756; doi:10.1038/s41467-024-52575-0)
Supplement: Supplementary file 8 — Reporting Summary [file 41467_2024_52575_MOESM8_ESM.pdf]

nature portfolio

Corresponding author(s): Mark von Zastrow

Last updated by author(s): Aug 5, 2024

Reporting Summary

Nature Portfolio wishes to improve the reproducibility of the work that we publish. This form provides structure for consistency and transparency in reporting. For further information on Nature Portfolio policies, see our Editorial Policies and the Editorial Policy Checklist.

Statistics

For all statistical analyses, confirm that the following items are present in the figure legend, table legend, main text, or Methods section.

n/a	Confirmed	
☐	☒	The exact sample size (*n*) for each experimental group/condition, given as a discrete number and unit of measurement
☐	☒	A statement on whether measurements were taken from distinct samples or whether the same sample was measured repeatedly
☐	☒	The statistical test(s) used AND whether they are one- or two-sided<br>*Only common tests should be described solely by name; describe more complex techniques in the Methods section.*
☐	☒	A description of all covariates tested
☐	☒	A description of any assumptions or corrections, such as tests of normality and adjustment for multiple comparisons
☐	☒	A full description of the statistical parameters including central tendency (e.g. means) or other basic estimates (e.g. regression coefficient) AND variation (e.g. standard deviation) or associated estimates of uncertainty (e.g. confidence intervals)
☐	☒	For null hypothesis testing, the test statistic (e.g. *F*, *t*, *r*) with confidence intervals, effect sizes, degrees of freedom and *P* value noted<br>*Give P values as exact values whenever suitable.*
☒	☐	For Bayesian analysis, information on the choice of priors and Markov chain Monte Carlo settings
☒	☐	For hierarchical and complex designs, identification of the appropriate level for tests and full reporting of outcomes
☒	☐	Estimates of effect sizes (e.g. Cohen's *d*, Pearson's *r*), indicating how they were calculated

Our web collection on statistics for biologists contains articles on many of the points above.

Software and code

Policy information about availability of computer code

Data collection	NIS Elements (Nikon), Micro-manager
Data analysis	ImageJ/Fiji, Prism 10 (Graphpad), Matlab, Microsoft Excel, Imaris 10.1

For manuscripts utilizing custom algorithms or software that are central to the research but not yet described in published literature, software must be made available to editors and reviewers. We strongly encourage code deposition in a community repository (e.g. GitHub). See the Nature Portfolio guidelines for submitting code & software for further information.

Data

Policy information about availability of data

All manuscripts must include a data availability statement. This statement should provide the following information, where applicable:

- Accession codes, unique identifiers, or web links for publicly available datasets
- A description of any restrictions on data availability
- For clinical datasets or third party data, please ensure that the statement adheres to our policy

All raw images used in the figures and source data for all graphs are provided in the Source Data folder

Research involving human participants, their data, or biological material

Policy information about studies with human participants or human data. See also policy information about sex, gender (identity/presentation), and sexual orientation and race, ethnicity and racism.

Reporting on sex and gender	Not applicable.
Reporting on race, ethnicity, or other socially relevant groupings	Not applicable.
Population characteristics	Not applicable.
Recruitment	Not applicable.
Ethics oversight	Not applicable.

Note that full information on the approval of the study protocol must also be provided in the manuscript.

Field-specific reporting

Please select the one below that is the best fit for your research. If you are not sure, read the appropriate sections before making your selection.

☒ Life sciences ☐ Behavioural & social sciences ☐ Ecological, evolutionary & environmental sciences

For a reference copy of the document with all sections, see nature.com/documents/nr-reporting-summary-flat.pdf

Life sciences study design

All studies must disclose on these points even when the disclosure is negative.

Sample size	No sample size calculation was performed. A minimum of three independent biological replicates was tested, representing cultures prepared from three independent animals.
Data exclusions	No data were excluded from analysis.
Replication	All experiments were carried out at least three times, and all findings in the manuscript were reproducible.
Randomization	No randomization was performed.
Blinding	Blinding is not relevant as most of the data collection and analysis methods are not vulnerable to human bias.

Reporting for specific materials, systems and methods

We require information from authors about some types of materials, experimental systems and methods used in many studies. Here, indicate whether each material, system or method listed is relevant to your study. If you are not sure if a list item applies to your research, read the appropriate section before selecting a response.

Materials & experimental systems

n/a	Involved in the study	
☐	☒	Antibodies
☐	☒	Eukaryotic cell lines
☒	☐	Palaeontology and archaeology
☐	☒	Animals and other organisms
☒	☐	Clinical data
☒	☐	Dual use research of concern
☒	☐	Plants

Methods

n/a	Involved in the study	
☒	☐	ChIP-seq
☒	☐	Flow cytometry
☒	☐	MRI-based neuroimaging

Antibodies

Antibodies used	Mouse anti-FLAG M1 (Millipore Sigma F3040), Rabbit anti PKAα cat (Santa Cruz sc-903), Mouse anti-PKA RIIβ (BD Biosciences 610625), Mouse anti-Giantin (Abcam ab37266), Rabbit anti-Arl13b (Proteintech 17711-1-AP), Rat anti-HA (BioLegend 901513), Mouse anti-EEA1 (BD Biosciences 610457), Goat anti-Vps35 (Novus Biologicals NB100-1397), Donkey anti rat IgG AlexaFluor 488

	(Invitrogen A-21208), Goat anti rat IgG AlexaFluor 488 (Invitrogen A-11006), Goat anti rat IgG AlexaFluor 647 (Invitrogen A-21247), Goat anti rabbit IgG AlexaFluor 647 (Invitrogen A-21245), Donkey anti mouse IgG AlexaFluor 555 (Invitrogen A-31570), Donkey anti mouse IgG AlexaFluor 647 (Invitrogen A-31571), Donkey anti goat IgG AlexaFluor 647 (Invitrogen A-21447), Donkey anti rabbit IgG AlexaFluor 555 (Invitrogen A-31572)
Validation	All antibodies are validated by the manufacturer, have been used in multiple publications and are routinely used in our laboratory.

Eukaryotic cell lines

Policy information about cell lines and Sex and Gender in Research

Cell line source(s)	HEK293 cells were used for adenylyl cyclase activity assays.
Authentication	ATCC validated cells (CRL-1573) were used
Mycoplasma contamination	We routinely test to verify lack of mycoplasma contamination
Commonly misidentified lines (See ICLAC register)	No commonly misidentified cells were used, per checking the ICLAC register

Animals and other research organisms

Policy information about studies involving animals; ARRIVE guidelines recommended for reporting animal research, and Sex and Gender in Research

Laboratory animals	E18 embryos were harvested from timed pregnant female Sprague-Dawley rats (Charles River)
Wild animals	Not applicable
Reporting on sex	Cultures were prepared from all embryos irrespective of sex
Field-collected samples	Not applicable
Ethics oversight	All procedures were performed according to the National Institutes of Health Guide for Care and Use of Laboratory Animals and approved by the UCSF Institutional Animal Care and Use Committee (protocol number AN185688)

Note that full information on the approval of the study protocol must also be provided in the manuscript.

Plants

Seed stocks	Not applicable
Novel plant genotypes	Not applicable
Authentication	Not applicable
