# Supplementary figures and images for: Spatial organization of adenylyl cyclase and its impact on dopamine signaling in neurons (part 2 of 2)
Source: Nat Commun. 2024 Sep 27;15:8297. doi: 10.1038/s41467-024-52575-0 (PMC11436756; doi:10.1038/s41467-024-52575-0)

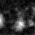

Supplement: Supplementary file 9 — Source data [file 41467_2024_52575_MOESM9_ESM.zip › Source Data/Fig 6/Fig 6e_right-panel_AC9-HaloTag_10min.tif]

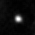

Supplement: Supplementary file 9 — Source data [file 41467_2024_52575_MOESM9_ESM.zip › Source Data/Fig 6/Fig 6e_right-panel_merge_6.7min.tif]

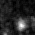

Supplement: Supplementary file 9 — Source data [file 41467_2024_52575_MOESM9_ESM.zip › Source Data/Fig 6/Fig 6e_right-panel_AC9-HaloTag_13.3min.tif]

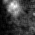

Supplement: Supplementary file 9 — Source data [file 41467_2024_52575_MOESM9_ESM.zip › Source Data/Fig 6/Fig 6e_right-panel_PKAcat-GFP_23.3min.tif]

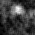

Supplement: Supplementary file 9 — Source data [file 41467_2024_52575_MOESM9_ESM.zip › Source Data/Fig 6/Fig 6e_right-panel_PKAcat-GFP_16.7min.tif]

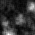

Supplement: Supplementary file 9 — Source data [file 41467_2024_52575_MOESM9_ESM.zip › Source Data/Fig 6/Fig 6e_right-panel_AC9-HaloTag_20min.tif]

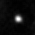

Supplement: Supplementary file 9 — Source data [file 41467_2024_52575_MOESM9_ESM.zip › Source Data/Fig 6/Fig 6e_right-panel_PKAcat-GFP_6.7min.tif]

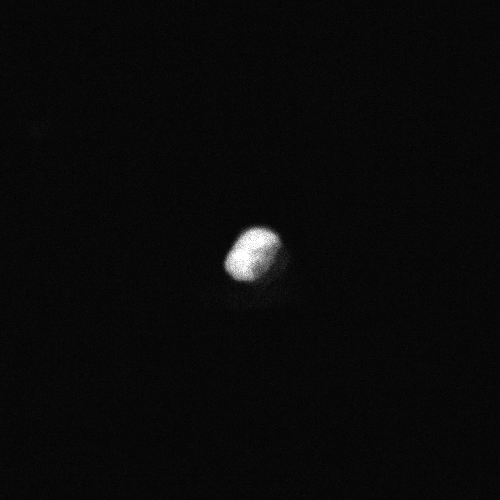

Supplement: Supplementary file 9 — Source data [file 41467_2024_52575_MOESM9_ESM.zip › Source Data/Fig 6/Fig 6g_ExRai-AKAR2-NLS.tif]

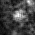

Supplement: Supplementary file 9 — Source data [file 41467_2024_52575_MOESM9_ESM.zip › Source Data/Fig 6/Fig 6e_right-panel_AC9-HaloTag_26.7min.tif]

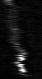

Supplement: Supplementary file 9 — Source data [file 41467_2024_52575_MOESM9_ESM.zip › Source Data/Fig 6/Fig 6f_Kymograph.tif]

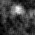

Supplement: Supplementary file 9 — Source data [file 41467_2024_52575_MOESM9_ESM.zip › Source Data/Fig 6/Fig 6e_right-panel_merge_16.7min.tif]

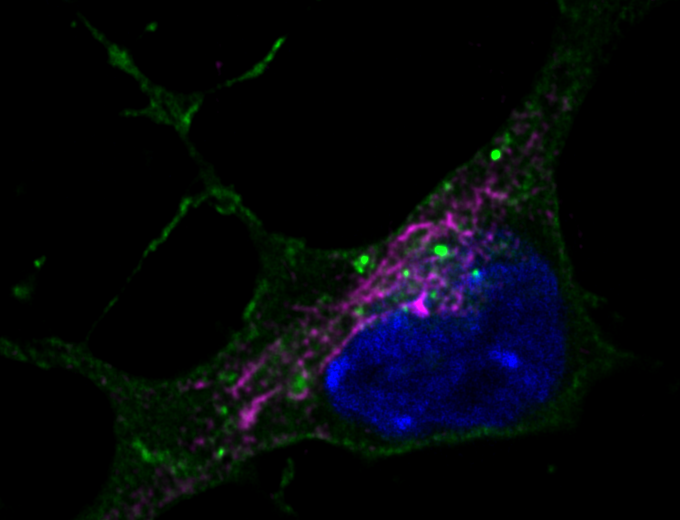

Supplement: Supplementary file 9 — Source data [file 41467_2024_52575_MOESM9_ESM.zip › Source Data/Fig 6/Fig 6c_merge.tif]

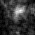

Supplement: Supplementary file 9 — Source data [file 41467_2024_52575_MOESM9_ESM.zip › Source Data/Fig 6/Fig 6e_right-panel_AC9-HaloTag_30min.tif]

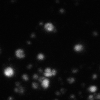

Supplement: Supplementary file 9 — Source data [file 41467_2024_52575_MOESM9_ESM.zip › Source Data/Fig 1/Fig 1g_crop_EEA1.tif]

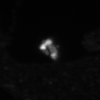

Supplement: Supplementary file 9 — Source data [file 41467_2024_52575_MOESM9_ESM.zip › Source Data/Fig 1/Fig 1a_crop_merge.tif]

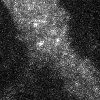

Supplement: Supplementary file 9 — Source data [file 41467_2024_52575_MOESM9_ESM.zip › Source Data/Fig 1/Fig 1f_crop_merge.tif]

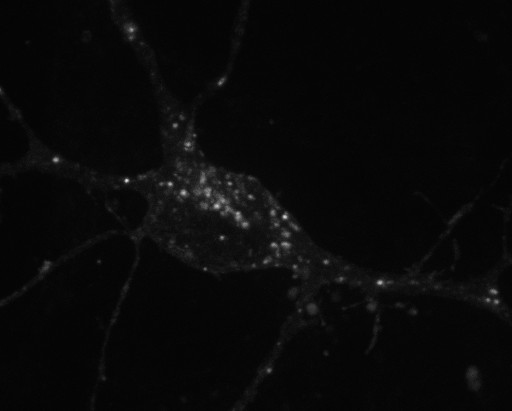

Supplement: Supplementary file 9 — Source data [file 41467_2024_52575_MOESM9_ESM.zip › Source Data/Fig 1/Fig 1h_AC9-GFP.tif]

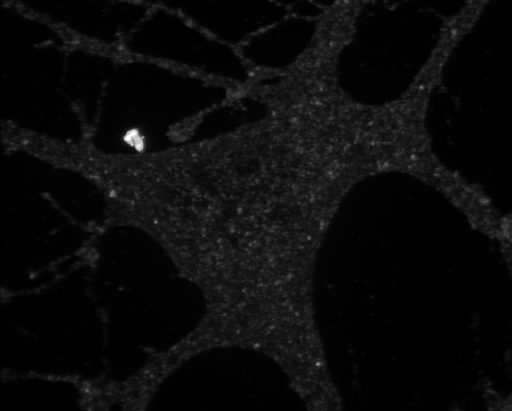

Supplement: Supplementary file 9 — Source data [file 41467_2024_52575_MOESM9_ESM.zip › Source Data/Fig 1/Fig 1a_HA-AC3.tif]

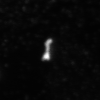

Supplement: Supplementary file 9 — Source data [file 41467_2024_52575_MOESM9_ESM.zip › Source Data/Fig 1/Fig 1c_crop_Arl13b.tif]

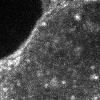

Supplement: Supplementary file 9 — Source data [file 41467_2024_52575_MOESM9_ESM.zip › Source Data/Fig 1/Fig 1g_crop_merge.tif]

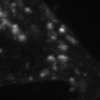

Supplement: Supplementary file 9 — Source data [file 41467_2024_52575_MOESM9_ESM.zip › Source Data/Fig 1/Fig 1h_crop_merge.tif]

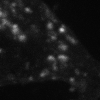

Supplement: Supplementary file 9 — Source data [file 41467_2024_52575_MOESM9_ESM.zip › Source Data/Fig 1/Fig 1h_crop_AC9-GFP.tif]

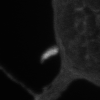

Supplement: Supplementary file 9 — Source data [file 41467_2024_52575_MOESM9_ESM.zip › Source Data/Fig 1/Fig 1b_crop_merge.tif]

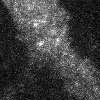

Supplement: Supplementary file 9 — Source data [file 41467_2024_52575_MOESM9_ESM.zip › Source Data/Fig 1/Fig 1f_crop_AC3-GFP.tif]

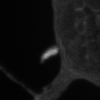

Supplement: Supplementary file 9 — Source data [file 41467_2024_52575_MOESM9_ESM.zip › Source Data/Fig 1/Fig 1b_crop_HA-AC5.tif]

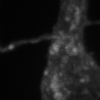

Supplement: Supplementary file 9 — Source data [file 41467_2024_52575_MOESM9_ESM.zip › Source Data/Fig 1/Fig 1c_crop_merge.tif]

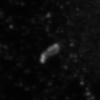

Supplement: Supplementary file 9 — Source data [file 41467_2024_52575_MOESM9_ESM.zip › Source Data/Fig 1/Fig 1b_crop_Arl13b.tif]

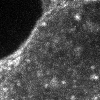

Supplement: Supplementary file 9 — Source data [file 41467_2024_52575_MOESM9_ESM.zip › Source Data/Fig 1/Fig 1g_crop_AC5-GFP.tif]

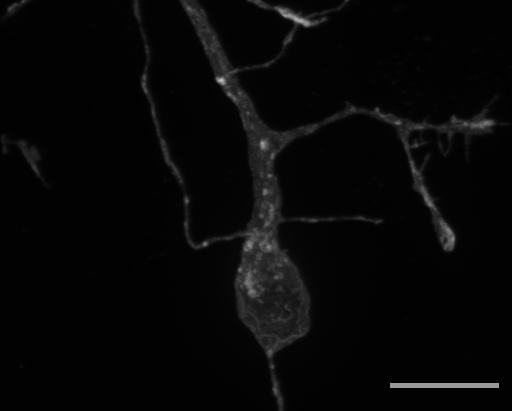

Supplement: Supplementary file 9 — Source data [file 41467_2024_52575_MOESM9_ESM.zip › Source Data/Fig 1/Fig 1c_HA-AC9.tif]

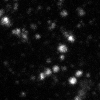

Supplement: Supplementary file 9 — Source data [file 41467_2024_52575_MOESM9_ESM.zip › Source Data/Fig 1/Fig 1h_crop_EEA1.tif]

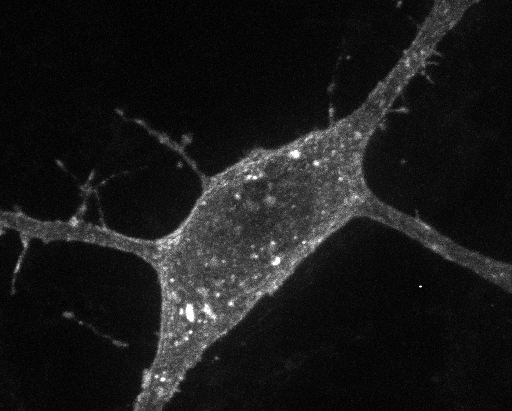

Supplement: Supplementary file 9 — Source data [file 41467_2024_52575_MOESM9_ESM.zip › Source Data/Fig 1/Fig 1g_AC5-GFP.tif]

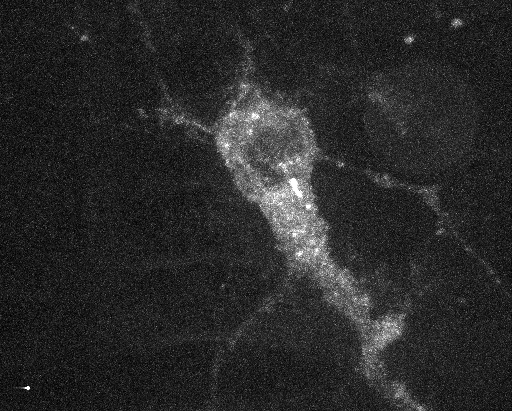

Supplement: Supplementary file 9 — Source data [file 41467_2024_52575_MOESM9_ESM.zip › Source Data/Fig 1/Fig 1f_AC3-GFP.tif]

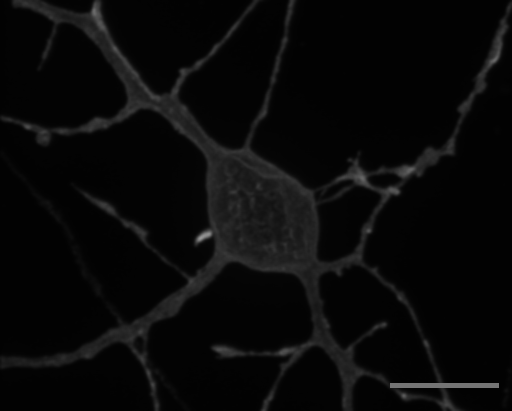

Supplement: Supplementary file 9 — Source data [file 41467_2024_52575_MOESM9_ESM.zip › Source Data/Fig 1/Fig 1b_HA-AC5.tif]

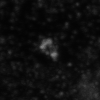

Supplement: Supplementary file 9 — Source data [file 41467_2024_52575_MOESM9_ESM.zip › Source Data/Fig 1/Fig 1a_crop_Arl13b.tif]

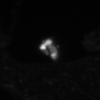

Supplement: Supplementary file 9 — Source data [file 41467_2024_52575_MOESM9_ESM.zip › Source Data/Fig 1/Fig 1a_crop_HA-AC3.tif]

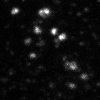

Supplement: Supplementary file 9 — Source data [file 41467_2024_52575_MOESM9_ESM.zip › Source Data/Fig 1/Fig 1f_crop_EEA1.tif]

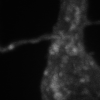

Supplement: Supplementary file 9 — Source data [file 41467_2024_52575_MOESM9_ESM.zip › Source Data/Fig 1/Fig 1c_crop_HA-AC9.tif]
