# Supplementary figures and images for: Spatial organization of adenylyl cyclase and its impact on dopamine signaling in neurons (part 1 of 2)
Source: Nat Commun. 2024 Sep 27;15:8297. doi: 10.1038/s41467-024-52575-0 (PMC11436756; doi:10.1038/s41467-024-52575-0)

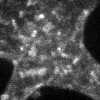

Supplement: Supplementary file 9 — Source data [file 41467_2024_52575_MOESM9_ESM.zip › Source Data/Fig 4/Fig 4g_left-panel_crop_AC5-AC9-1-35-GFP.tif]

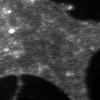

Supplement: Supplementary file 9 — Source data [file 41467_2024_52575_MOESM9_ESM.zip › Source Data/Fig 4/Fig 4i_right-panel_crop_merge.tif]

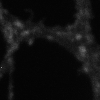

Supplement: Supplementary file 9 — Source data [file 41467_2024_52575_MOESM9_ESM.zip › Source Data/Fig 4/Fig 4b_crop_AC5-AC9-Nter-GFP.tif]

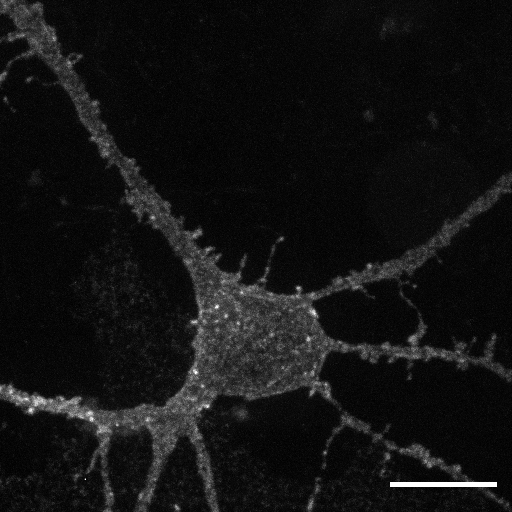

Supplement: Supplementary file 9 — Source data [file 41467_2024_52575_MOESM9_ESM.zip › Source Data/Fig 4/Fig 4g_right-panel_AC5-AC9-1-35-LL>AA-GFP.tif]

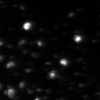

Supplement: Supplementary file 9 — Source data [file 41467_2024_52575_MOESM9_ESM.zip › Source Data/Fig 4/Fig 4i_left-panel_crop_EEA1.tif]

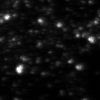

Supplement: Supplementary file 9 — Source data [file 41467_2024_52575_MOESM9_ESM.zip › Source Data/Fig 4/Fig 4i_right-panel_crop_EEA1.tif]

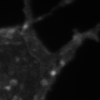

Supplement: Supplementary file 9 — Source data [file 41467_2024_52575_MOESM9_ESM.zip › Source Data/Fig 4/Fig 4i_left-panel_crop_merge.tif]

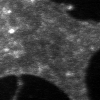

Supplement: Supplementary file 9 — Source data [file 41467_2024_52575_MOESM9_ESM.zip › Source Data/Fig 4/Fig 4i_right-panel_crop_AC9-LL>AA-GFP.tif]

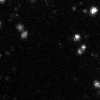

Supplement: Supplementary file 9 — Source data [file 41467_2024_52575_MOESM9_ESM.zip › Source Data/Fig 4/Fig 4b_crop_EEA1.tif]

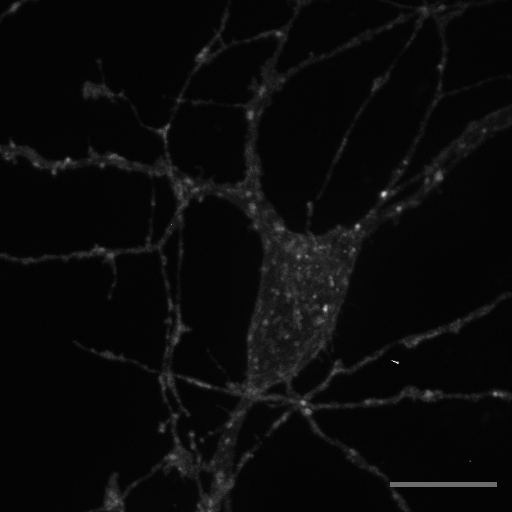

Supplement: Supplementary file 9 — Source data [file 41467_2024_52575_MOESM9_ESM.zip › Source Data/Fig 4/Fig 4b_AC5-AC9-Nter-GFP.tif]

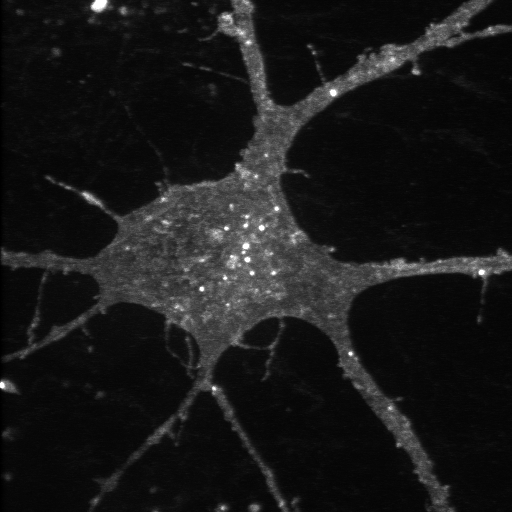

Supplement: Supplementary file 9 — Source data [file 41467_2024_52575_MOESM9_ESM.zip › Source Data/Fig 4/Fig 4i_right-panel_AC9-LL>AA-GFP.tif]

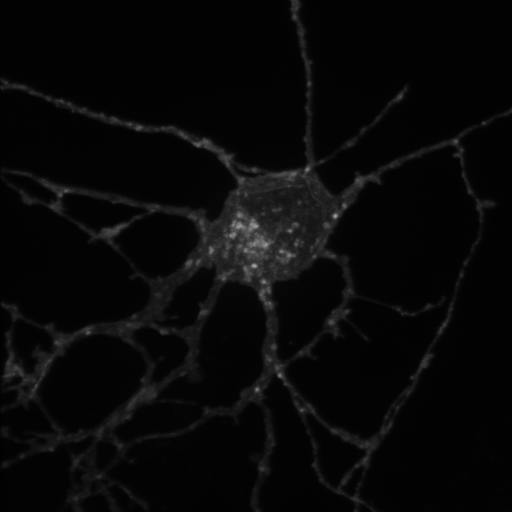

Supplement: Supplementary file 9 — Source data [file 41467_2024_52575_MOESM9_ESM.zip › Source Data/Fig 4/Fig 4i_left-panel_AC9-GFP.tif]

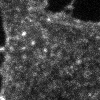

Supplement: Supplementary file 9 — Source data [file 41467_2024_52575_MOESM9_ESM.zip › Source Data/Fig 4/Fig 4g_right-panel_crop_AC5-AC9-1-35-LL>AA-GFP.tif]

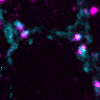

Supplement: Supplementary file 9 — Source data [file 41467_2024_52575_MOESM9_ESM.zip › Source Data/Fig 4/Fig 4b_crop_merge.tif]

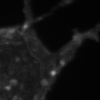

Supplement: Supplementary file 9 — Source data [file 41467_2024_52575_MOESM9_ESM.zip › Source Data/Fig 4/Fig 4i_left-panel_crop_AC9-GFP.tif]

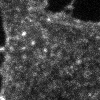

Supplement: Supplementary file 9 — Source data [file 41467_2024_52575_MOESM9_ESM.zip › Source Data/Fig 4/Fig 4g_right-panel_crop_merge.tif]

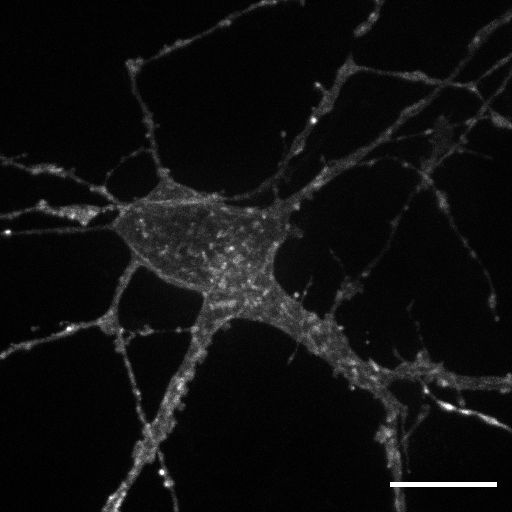

Supplement: Supplementary file 9 — Source data [file 41467_2024_52575_MOESM9_ESM.zip › Source Data/Fig 4/Fig 4g_left-panel_AC5-AC9-1-35-GFP.tif]

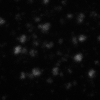

Supplement: Supplementary file 9 — Source data [file 41467_2024_52575_MOESM9_ESM.zip › Source Data/Fig 4/Fig 4g_left-panel_crop_EEA1.tif]

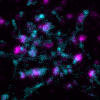

Supplement: Supplementary file 9 — Source data [file 41467_2024_52575_MOESM9_ESM.zip › Source Data/Fig 4/Fig 4g_left-panel_crop_merge.tif]

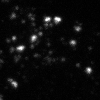

Supplement: Supplementary file 9 — Source data [file 41467_2024_52575_MOESM9_ESM.zip › Source Data/Fig 4/Fig 4g_right-panel_crop_EEA1.tif]

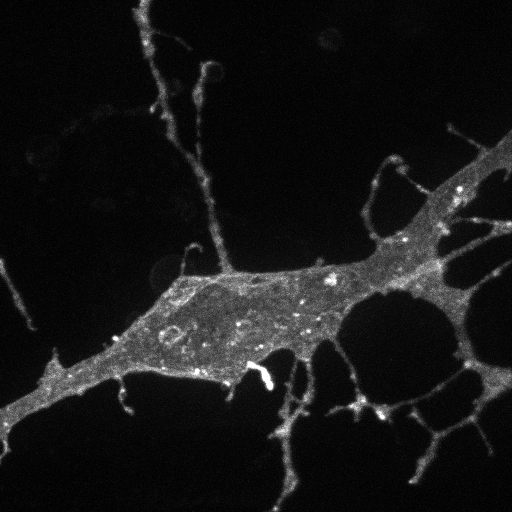

Supplement: Supplementary file 9 — Source data [file 41467_2024_52575_MOESM9_ESM.zip › Source Data/Fig 3/Fig 3c_left-panel_AC5-GFP.tif]

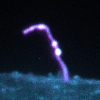

Supplement: Supplementary file 9 — Source data [file 41467_2024_52575_MOESM9_ESM.zip › Source Data/Fig 3/Fig 3a_left-panel_crop_merge.tif]

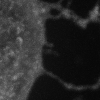

Supplement: Supplementary file 9 — Source data [file 41467_2024_52575_MOESM9_ESM.zip › Source Data/Fig 3/Fig 3c_right-panel_crop_AC5-dNter-GFP.tif]

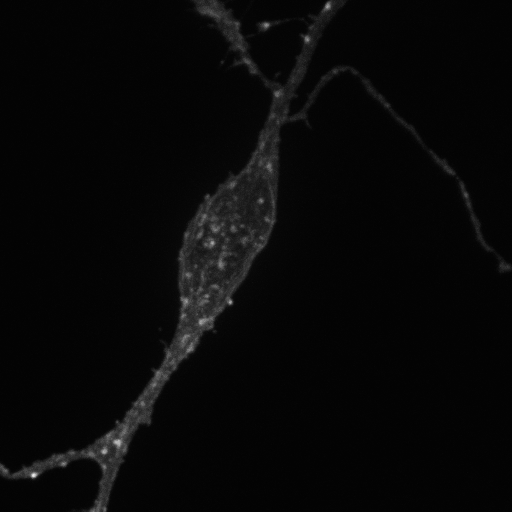

Supplement: Supplementary file 9 — Source data [file 41467_2024_52575_MOESM9_ESM.zip › Source Data/Fig 3/Fig 3a_right-panel_AC5-AC9-Nter-GFP.tif]

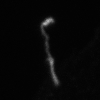

Supplement: Supplementary file 9 — Source data [file 41467_2024_52575_MOESM9_ESM.zip › Source Data/Fig 3/Fig 3a_right-panel_crop_FLAG-D1R.tif]

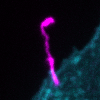

Supplement: Supplementary file 9 — Source data [file 41467_2024_52575_MOESM9_ESM.zip › Source Data/Fig 3/Fig 3a_right-panel_crop_merge.tif]

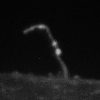

Supplement: Supplementary file 9 — Source data [file 41467_2024_52575_MOESM9_ESM.zip › Source Data/Fig 3/Fig 3a_left-panel_crop_AC5-GFP.tif]

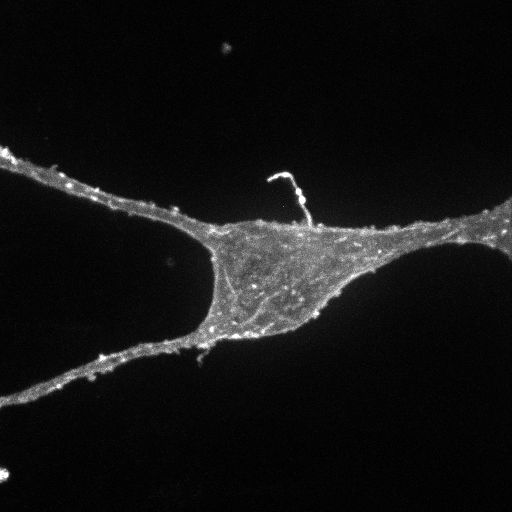

Supplement: Supplementary file 9 — Source data [file 41467_2024_52575_MOESM9_ESM.zip › Source Data/Fig 3/Fig 3a_left-panel_AC5-GFP.tif]

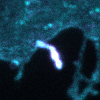

Supplement: Supplementary file 9 — Source data [file 41467_2024_52575_MOESM9_ESM.zip › Source Data/Fig 3/Fig 3c_left-panel_crop_merge.tif]

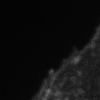

Supplement: Supplementary file 9 — Source data [file 41467_2024_52575_MOESM9_ESM.zip › Source Data/Fig 3/Fig 3a_right-panel_crop_AC5-AC9-Nter-GFP.tif]

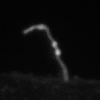

Supplement: Supplementary file 9 — Source data [file 41467_2024_52575_MOESM9_ESM.zip › Source Data/Fig 3/Fig 3a_left-panel_crop_FLAG-D1R.tif]

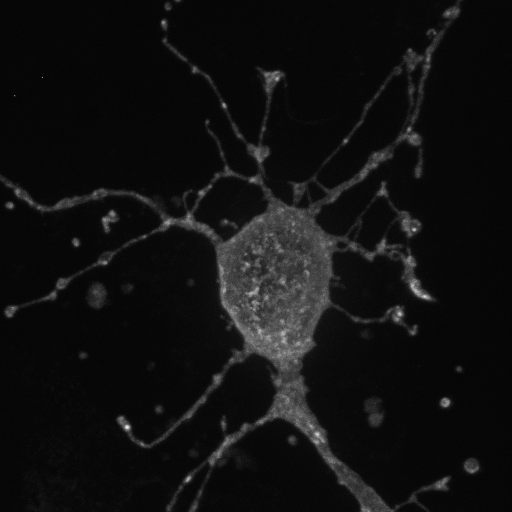

Supplement: Supplementary file 9 — Source data [file 41467_2024_52575_MOESM9_ESM.zip › Source Data/Fig 3/Fig 3c_right-panel_AC5-dNter-GFP.tif]

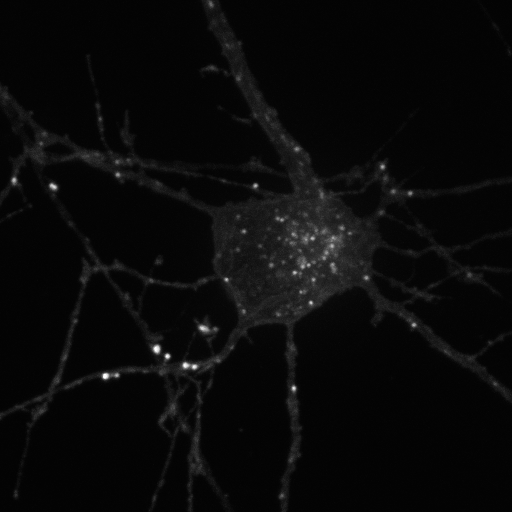

Supplement: Supplementary file 9 — Source data [file 41467_2024_52575_MOESM9_ESM.zip › Source Data/Fig 3/Fig 3e_left-panel_AC9-GFP.tif]

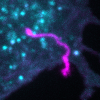

Supplement: Supplementary file 9 — Source data [file 41467_2024_52575_MOESM9_ESM.zip › Source Data/Fig 3/Fig 3e_left-panel_crop_merge.tif]

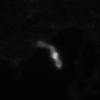

Supplement: Supplementary file 9 — Source data [file 41467_2024_52575_MOESM9_ESM.zip › Source Data/Fig 3/Fig 3c_left-panel_crop_FLAG-D1R.tif]

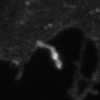

Supplement: Supplementary file 9 — Source data [file 41467_2024_52575_MOESM9_ESM.zip › Source Data/Fig 3/Fig 3c_left-panel_crop_AC5-GFP.tif]

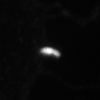

Supplement: Supplementary file 9 — Source data [file 41467_2024_52575_MOESM9_ESM.zip › Source Data/Fig 3/Fig 3c_right-panel_crop_FLAG-D1R.tif]

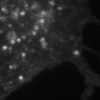

Supplement: Supplementary file 9 — Source data [file 41467_2024_52575_MOESM9_ESM.zip › Source Data/Fig 3/Fig 3e_left-panel_crop_AC9-GFP.tif]

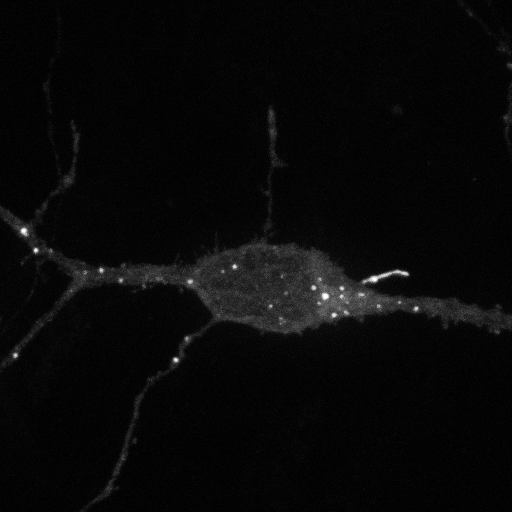

Supplement: Supplementary file 9 — Source data [file 41467_2024_52575_MOESM9_ESM.zip › Source Data/Fig 3/Fig 3e_right-panel_AC9-AC5-Nter-GFP.tif]

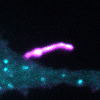

Supplement: Supplementary file 9 — Source data [file 41467_2024_52575_MOESM9_ESM.zip › Source Data/Fig 3/Fig 3e_right-panel_crop_merge.tif]

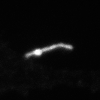

Supplement: Supplementary file 9 — Source data [file 41467_2024_52575_MOESM9_ESM.zip › Source Data/Fig 3/Fig 3e_right-panel_crop_FLAG-D1R.tif]

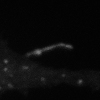

Supplement: Supplementary file 9 — Source data [file 41467_2024_52575_MOESM9_ESM.zip › Source Data/Fig 3/Fig 3e_right-panel_crop_AC9-AC5-Nter-GFP.tif]

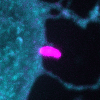

Supplement: Supplementary file 9 — Source data [file 41467_2024_52575_MOESM9_ESM.zip › Source Data/Fig 3/Fig 3c_right-panel_crop_merge.tif]

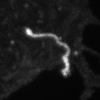

Supplement: Supplementary file 9 — Source data [file 41467_2024_52575_MOESM9_ESM.zip › Source Data/Fig 3/Fig 3e_left-panel_crop_FLAG-D1R.tif]

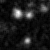

Supplement: Supplementary file 9 — Source data [file 41467_2024_52575_MOESM9_ESM.zip › Source Data/Fig 2/Fig 2c_crop_merge_30min.tif]

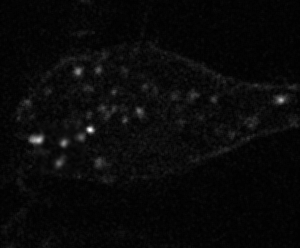

Supplement: Supplementary file 9 — Source data [file 41467_2024_52575_MOESM9_ESM.zip › Source Data/Fig 2/Fig 2c_merge_20min.tif]

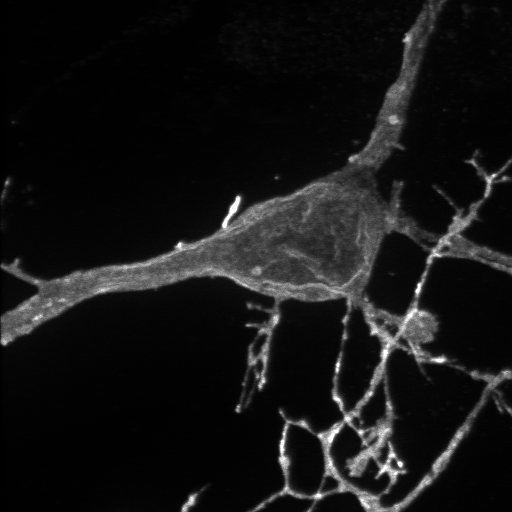

Supplement: Supplementary file 9 — Source data [file 41467_2024_52575_MOESM9_ESM.zip › Source Data/Fig 2/Fig 2b_merge.tif]

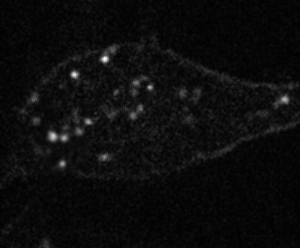

Supplement: Supplementary file 9 — Source data [file 41467_2024_52575_MOESM9_ESM.zip › Source Data/Fig 2/Fig 2c_merge_30min.tif]

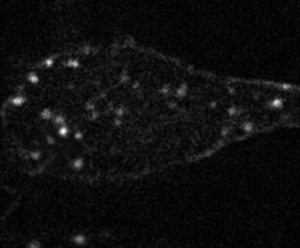

Supplement: Supplementary file 9 — Source data [file 41467_2024_52575_MOESM9_ESM.zip › Source Data/Fig 2/Fig 2c_AC9-GFP_0min.tif]

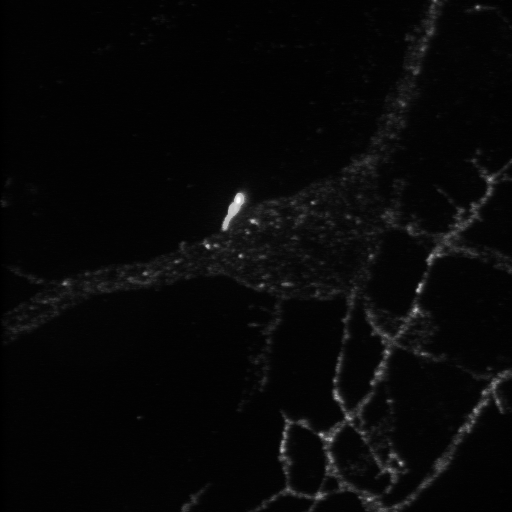

Supplement: Supplementary file 9 — Source data [file 41467_2024_52575_MOESM9_ESM.zip › Source Data/Fig 2/Fig 2b_FLAG-D1R.tif]

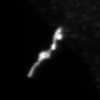

Supplement: Supplementary file 9 — Source data [file 41467_2024_52575_MOESM9_ESM.zip › Source Data/Fig 2/Fig 2a_crop_HA-AC3.tif]

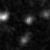

Supplement: Supplementary file 9 — Source data [file 41467_2024_52575_MOESM9_ESM.zip › Source Data/Fig 2/Fig 2c_crop_merge_20min.tif]

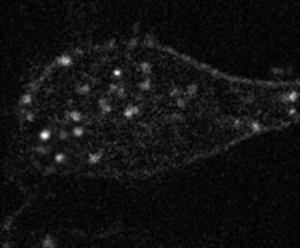

Supplement: Supplementary file 9 — Source data [file 41467_2024_52575_MOESM9_ESM.zip › Source Data/Fig 2/Fig 2c_merge_10min.tif]

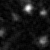

Supplement: Supplementary file 9 — Source data [file 41467_2024_52575_MOESM9_ESM.zip › Source Data/Fig 2/Fig 2c_crop_AC9-GFP_0min.tif]

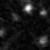

Supplement: Supplementary file 9 — Source data [file 41467_2024_52575_MOESM9_ESM.zip › Source Data/Fig 2/Fig 2c_crop_merge_0min.tif]

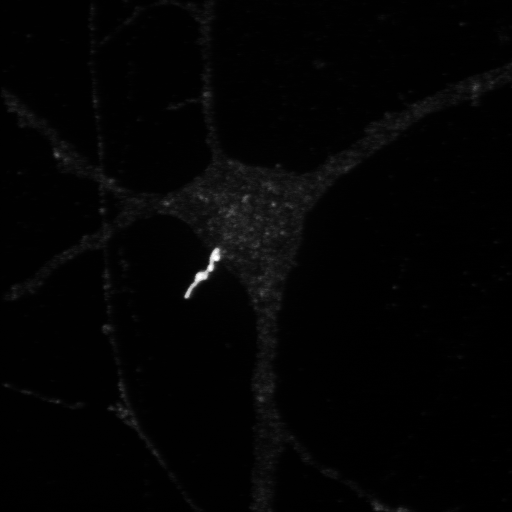

Supplement: Supplementary file 9 — Source data [file 41467_2024_52575_MOESM9_ESM.zip › Source Data/Fig 2/Fig 2a_HA-AC3.tif]

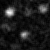

Supplement: Supplementary file 9 — Source data [file 41467_2024_52575_MOESM9_ESM.zip › Source Data/Fig 2/Fig 2c_crop_merge_10min.tif]

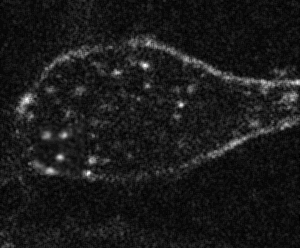

Supplement: Supplementary file 9 — Source data [file 41467_2024_52575_MOESM9_ESM.zip › Source Data/Fig 2/Fig 2c_FLAG-D1R_10min.tif]

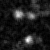

Supplement: Supplementary file 9 — Source data [file 41467_2024_52575_MOESM9_ESM.zip › Source Data/Fig 2/Fig 2c_crop_FLAG-D1R_30min.tif]

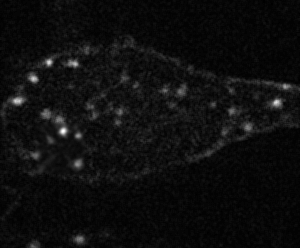

Supplement: Supplementary file 9 — Source data [file 41467_2024_52575_MOESM9_ESM.zip › Source Data/Fig 2/Fig 2c_merge_0min.tif]

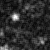

Supplement: Supplementary file 9 — Source data [file 41467_2024_52575_MOESM9_ESM.zip › Source Data/Fig 2/Fig 2c_crop_FLAG-D1R_0min.tif]

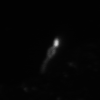

Supplement: Supplementary file 9 — Source data [file 41467_2024_52575_MOESM9_ESM.zip › Source Data/Fig 2/Fig 2b_crop_FLAG-D1R.tif]

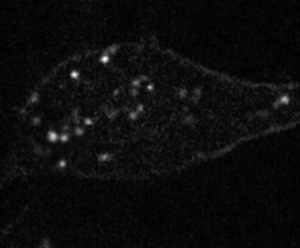

Supplement: Supplementary file 9 — Source data [file 41467_2024_52575_MOESM9_ESM.zip › Source Data/Fig 2/Fig 2c_AC9-GFP_30min.tif]

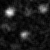

Supplement: Supplementary file 9 — Source data [file 41467_2024_52575_MOESM9_ESM.zip › Source Data/Fig 2/Fig 2c_crop_AC9-GFP_10min.tif]

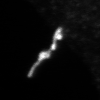

Supplement: Supplementary file 9 — Source data [file 41467_2024_52575_MOESM9_ESM.zip › Source Data/Fig 2/Fig 2a_crop_merge.tif]

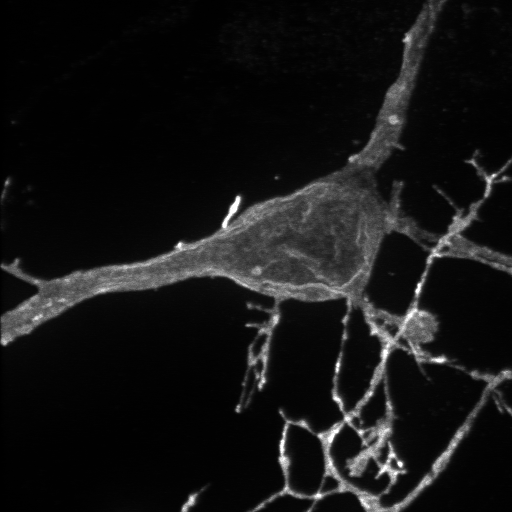

Supplement: Supplementary file 9 — Source data [file 41467_2024_52575_MOESM9_ESM.zip › Source Data/Fig 2/Fig 2b_HA-AC5.tif]

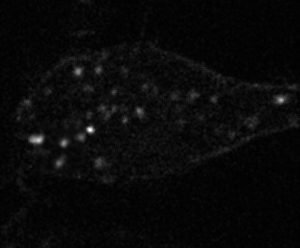

Supplement: Supplementary file 9 — Source data [file 41467_2024_52575_MOESM9_ESM.zip › Source Data/Fig 2/Fig 2c_AC9-GFP_20min.tif]

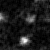

Supplement: Supplementary file 9 — Source data [file 41467_2024_52575_MOESM9_ESM.zip › Source Data/Fig 2/Fig 2c_crop_FLAG-D1R_20min.tif]

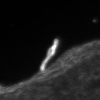

Supplement: Supplementary file 9 — Source data [file 41467_2024_52575_MOESM9_ESM.zip › Source Data/Fig 2/Fig 2b_crop_HA-AC5.tif]

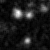

Supplement: Supplementary file 9 — Source data [file 41467_2024_52575_MOESM9_ESM.zip › Source Data/Fig 2/Fig 2c_crop_AC9-GFP_30min.tif]

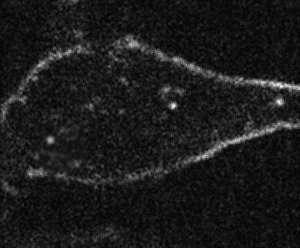

Supplement: Supplementary file 9 — Source data [file 41467_2024_52575_MOESM9_ESM.zip › Source Data/Fig 2/Fig 2c_FLAG-D1R_0min.tif]

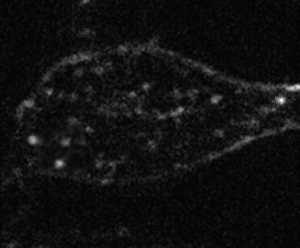

Supplement: Supplementary file 9 — Source data [file 41467_2024_52575_MOESM9_ESM.zip › Source Data/Fig 2/Fig 2c_FLAG-D1R_20min.tif]

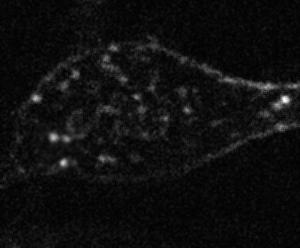

Supplement: Supplementary file 9 — Source data [file 41467_2024_52575_MOESM9_ESM.zip › Source Data/Fig 2/Fig 2c_FLAG-D1R_30min.tif]

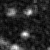

Supplement: Supplementary file 9 — Source data [file 41467_2024_52575_MOESM9_ESM.zip › Source Data/Fig 2/Fig 2c_crop_FLAG-D1R_10min.tif]

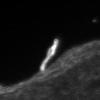

Supplement: Supplementary file 9 — Source data [file 41467_2024_52575_MOESM9_ESM.zip › Source Data/Fig 2/Fig 2b_crop_merge.tif]

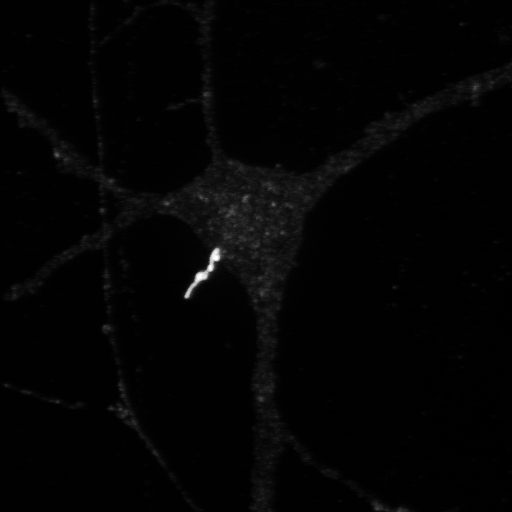

Supplement: Supplementary file 9 — Source data [file 41467_2024_52575_MOESM9_ESM.zip › Source Data/Fig 2/Fig 2a_merge.tif]

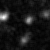

Supplement: Supplementary file 9 — Source data [file 41467_2024_52575_MOESM9_ESM.zip › Source Data/Fig 2/Fig 2c_crop_AC9-GFP_20min.tif]

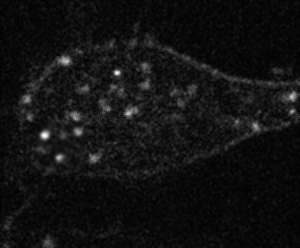

Supplement: Supplementary file 9 — Source data [file 41467_2024_52575_MOESM9_ESM.zip › Source Data/Fig 2/Fig 2c_AC9-GFP_10min.tif]

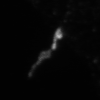

Supplement: Supplementary file 9 — Source data [file 41467_2024_52575_MOESM9_ESM.zip › Source Data/Fig 2/Fig 2a_crop_FLAG-D1R.tif]

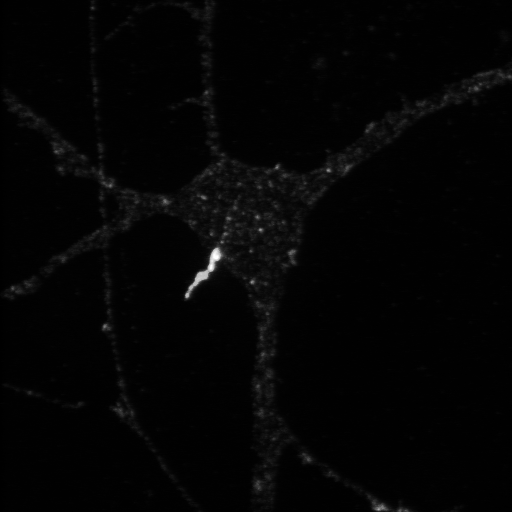

Supplement: Supplementary file 9 — Source data [file 41467_2024_52575_MOESM9_ESM.zip › Source Data/Fig 2/Fig 2a_FLAG-D1R.tif]

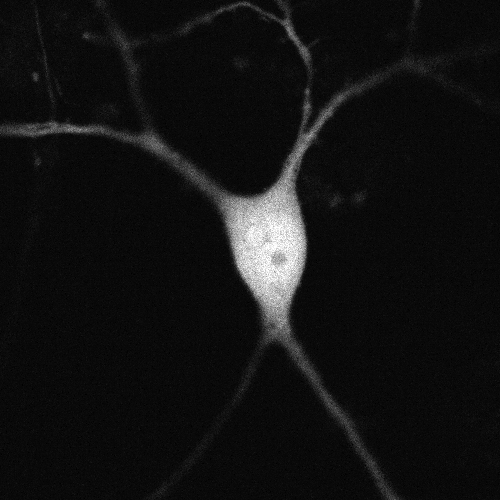

Supplement: Supplementary file 9 — Source data [file 41467_2024_52575_MOESM9_ESM.zip › Source Data/Fig 5/Fig 5d_cADDis.tif]

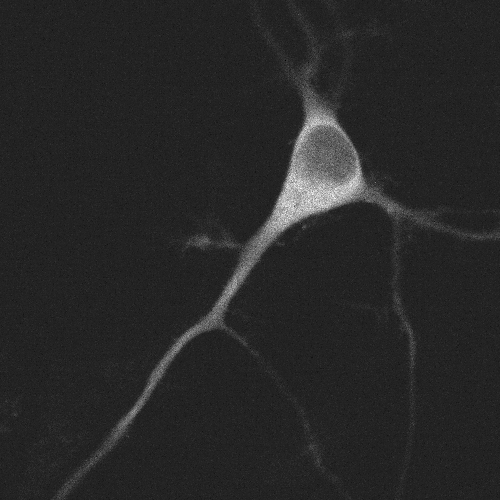

Supplement: Supplementary file 9 — Source data [file 41467_2024_52575_MOESM9_ESM.zip › Source Data/Fig 5/Fig 5d_ExRai-AKAR2.tif]

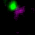

Supplement: Supplementary file 9 — Source data [file 41467_2024_52575_MOESM9_ESM.zip › Source Data/Fig 6/Fig 6e_right-panel_merge_30min.tif]

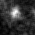

Supplement: Supplementary file 9 — Source data [file 41467_2024_52575_MOESM9_ESM.zip › Source Data/Fig 6/Fig 6e_right-panel_PKAcat-GFP_20min.tif]

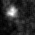

Supplement: Supplementary file 9 — Source data [file 41467_2024_52575_MOESM9_ESM.zip › Source Data/Fig 6/Fig 6e_right-panel_PKAcat-GFP_26.7min.tif]

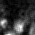

Supplement: Supplementary file 9 — Source data [file 41467_2024_52575_MOESM9_ESM.zip › Source Data/Fig 6/Fig 6e_right-panel_AC9-HaloTag_6.7min.tif]

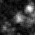

Supplement: Supplementary file 9 — Source data [file 41467_2024_52575_MOESM9_ESM.zip › Source Data/Fig 6/Fig 6e_right-panel_AC9-HaloTag_16.7min.tif]

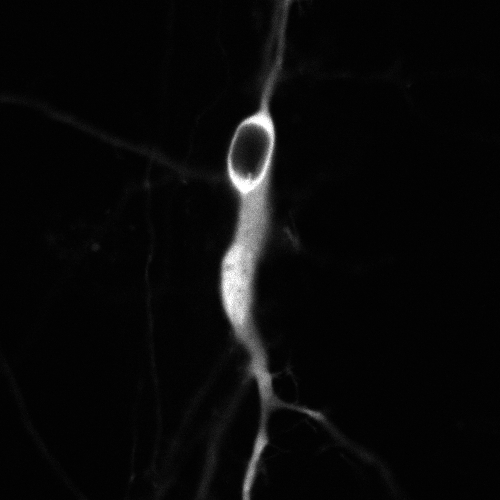

Supplement: Supplementary file 9 — Source data [file 41467_2024_52575_MOESM9_ESM.zip › Source Data/Fig 6/Fig 6j_ExRai-AKAR2-NES.tif]

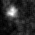

Supplement: Supplementary file 9 — Source data [file 41467_2024_52575_MOESM9_ESM.zip › Source Data/Fig 6/Fig 6e_right-panel_merge_26.7min.tif]

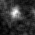

Supplement: Supplementary file 9 — Source data [file 41467_2024_52575_MOESM9_ESM.zip › Source Data/Fig 6/Fig 6e_right-panel_merge_20min.tif]

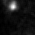

Supplement: Supplementary file 9 — Source data [file 41467_2024_52575_MOESM9_ESM.zip › Source Data/Fig 6/Fig 6e_right-panel_PKAcat-GFP_30min.tif]

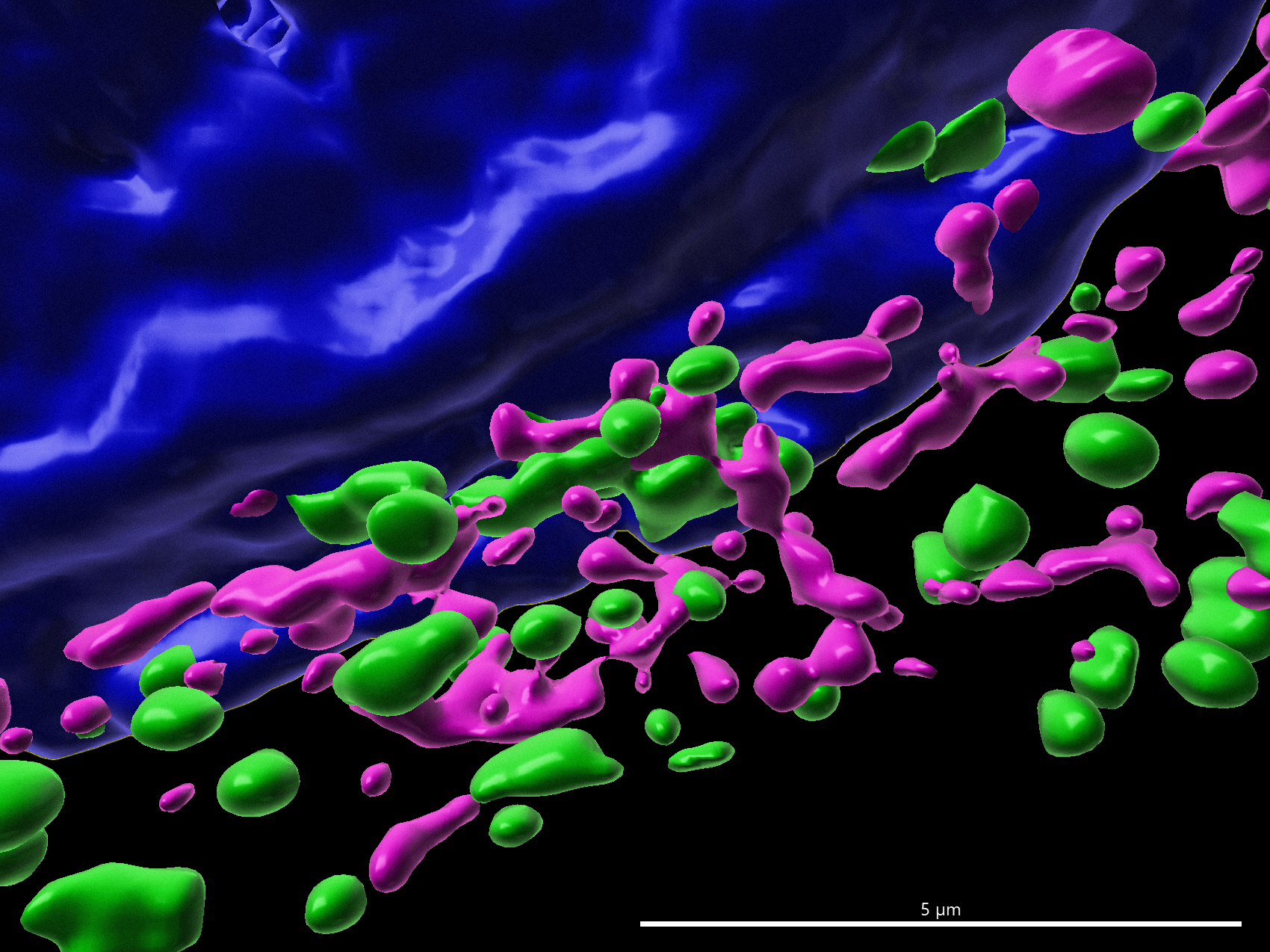

Supplement: Supplementary file 9 — Source data [file 41467_2024_52575_MOESM9_ESM.zip › Source Data/Fig 6/Fig 6b_3D-model.tif]

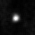

Supplement: Supplementary file 9 — Source data [file 41467_2024_52575_MOESM9_ESM.zip › Source Data/Fig 6/Fig 6e_right-panel_PKAcat-GFP_10min.tif]

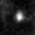

Supplement: Supplementary file 9 — Source data [file 41467_2024_52575_MOESM9_ESM.zip › Source Data/Fig 6/Fig 6e_right-panel_merge_13.3min.tif]

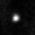

Supplement: Supplementary file 9 — Source data [file 41467_2024_52575_MOESM9_ESM.zip › Source Data/Fig 6/Fig 6e_right-panel_merge_10min.tif]

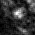

Supplement: Supplementary file 9 — Source data [file 41467_2024_52575_MOESM9_ESM.zip › Source Data/Fig 6/Fig 6e_right-panel_AC9-HaloTag_23.3min.tif]

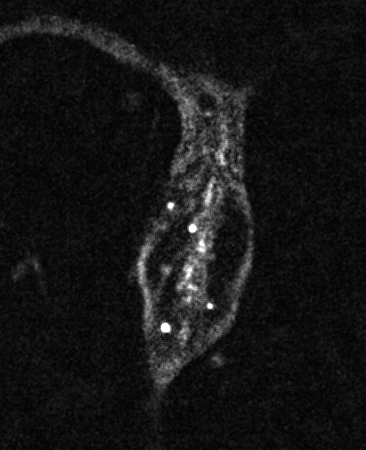

Supplement: Supplementary file 9 — Source data [file 41467_2024_52575_MOESM9_ESM.zip › Source Data/Fig 6/Fig 6e_left-panel_PKAcat-GFP_ADCY9-HaloTag.tif]

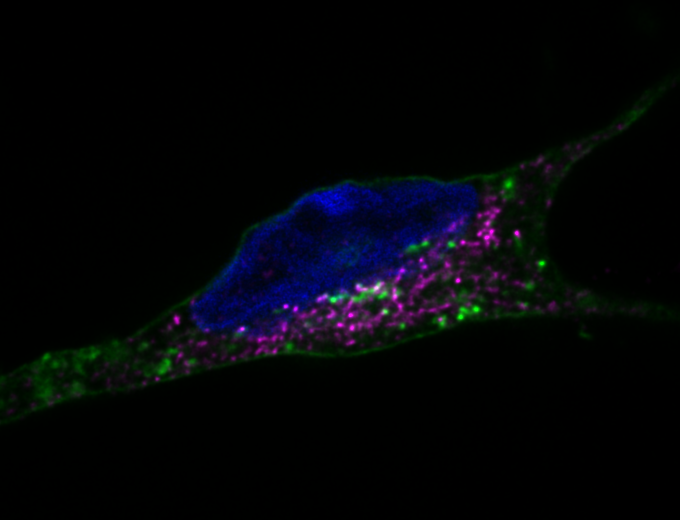

Supplement: Supplementary file 9 — Source data [file 41467_2024_52575_MOESM9_ESM.zip › Source Data/Fig 6/Fig 6a_merge.tif]

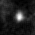

Supplement: Supplementary file 9 — Source data [file 41467_2024_52575_MOESM9_ESM.zip › Source Data/Fig 6/Fig 6e_right-panel_PKAcat-GFP_13.3min.tif]

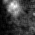

Supplement: Supplementary file 9 — Source data [file 41467_2024_52575_MOESM9_ESM.zip › Source Data/Fig 6/Fig 6e_right-panel_merge_23.3min.tif]
